# CD30 expression is a novel prognostic indicator in extranodal natural killer/T-cell lymphoma, nasal type

**DOI:** 10.1186/1471-2407-14-890

**Published:** 2014-11-28

**Authors:** Pengfei Li, Li Jiang, Xinke Zhang, Jun Liu, Hua Wang

**Affiliations:** State Key Laboratory of Oncology in South China, 651 Dongfeng East Road, Guangzhou, 510060 China; Department of Pathology, Sun Yat-Sen University Department of Pathology, Sun Yat-Sen University Cancer Center, 651 Dongfeng East Road, Guangzhou, 510060 Guangdong China; Collaborative Innovation Center of Cancer Medicine, Sun Yat-Sen University Cancer Center, Guangzhou, China

**Keywords:** Extranodal natural killer (NK)/T-cell lymphoma, nasal type (ENKTL), CD30, Immunohistochemistry, Prognosis

## Abstract

**Background:**

Extranodal natural killer/T-cell lymphoma, nasal type (ENKTL), is an aggressive type of lymphoma whose standard treatment and validated prognostic model have not yet been defined.

**Methods:**

CD30 expression was detected using immunohistochemistry in 96 ENKTL patients, and the data were used to evaluate its relationship with clinical features, treatment response and prognosis.

**Results:**

Expression of CD30 was detected in 31.2% of ENKTL patients, which was significantly correlated with B symptoms and elevated serum lactate dehydrogenase. The complete remission rate was not significantly different between CD30-positive and negative groups. After a median follow-up time of 31 months, 5-year overall survival (OS) and 5-year progression-free survival (PFS) rates in the CD30-positive group were both significantly lower than those in the CD30-negative group (34.1% vs. 64.4%, P = 0.002, for 5 year-OS; 26.0% vs. 66.7%, P < 0.001, for 5 year-PFS). In patients with an International Prognostic Index (IPI) or Korean Prognostic Index (KPI) score of 0–1, CD30 positivity was associated with shorter 5-year OS and PFS (IPI: P = 0.001 and 0.002, respectively; KPI: P = 0.018 and 0.023, respectively). In a multivariate Cox regression model, CD30 expression and stage were independent prognostic factors for OS (p = 0.004 and p = 0.012, respectively) and PFS (p = 0.001 and p = 0.022, respectively).

**Conclusions:**

Our results showed that expression of CD30 was not related to response to treatment but was an independent prognostic factor for both OS and PFS in ENKTL, nasal type, which suggests a role for CD30 in the pathogenesis of this disease and may support the incorporation of anti-CD30-targeted therapy into the treatment paradigm for ENKTL.

## Background

Extranodal natural killer (NK)/T-cell lymphoma (ENKTL), nasal type, is a distinct and heterogeneous histopathologic subtype of non-Hodgkin lymphoma (NHL) characterized by vascular damage and destruction, prominent necrosis and association with the Epstein-Barr virus (EBV) [1, 2]. There is an ethnic and geographical predisposition to ENKTL. Though uncommon in Western countries, ENKTL is relatively more common in Asia and Latin America [3, 4] and accounts for 5–10% of all malignant lymphomas in China. Owing to its poor prognosis, a great deal of clinical and pathological work has been undertaken to study prognostic markers in ENKTL.

Clinically, two major prognostic models have been applied to study NK/T-cell lymphomas: the International Prognostic Index (IPI; age, PS, stage, lactate dehydrogenase (LDH) level and extranodal sites) and the Korean Prognostic Index (KPI: stage, LDH level, B symptoms and regional lymph nodes). IPI has been widely used for both predicting prognosis and selecting therapeutic options in patients with aggressive NHL. However, its value has not been confirmed in ENKTL because almost 80% of patients with ENKTL were in the low IPI risk group (score 0–1), in which a good deal of heterogeneity exists. The KPI was developed in the era of anthracycline-based chemotherapy, which seems better than the IPI [5, 6]. However, the prognostic value of KPI could not be repeated in some studies, especially in the era of asparaginase-based chemotherapy [7], suggesting that both IPI and KPI scoring systems could be further improved. Moreover, the two prognostic models are based on clinical features before treatment; the pathological or molecular markers for predicting the outcome of ENKTL have not yet been well defined. Recently, some useful biomarkers have been found to be independent prognostic factors in ENKTL [8], such as serum levels of interleukin-9 [9] and serum C-reactive protein [10].

Recently, there have been some sporadic comparisons of the CD30 cDNA with known sequences indicating that the extracellular domain of CD30 is related to members of the tumor necrosis factor receptor (TNFR) superfamily, which includes TNFR-1, TNFR-2 and low-affinity nerve growth factor receptor [11]. The recently cloned membrane-bound CD30 ligand (CD30L) belongs to the TNF ligand superfamily and confirms that CD30 might act as a cytokine receptor [12]. Functional studies using recombinant CD30L showed proliferative effects on some HD-derived cell lines and a T-All cell line [13]. There are some scattered reports regarding CD30 expression in ENKTL patients. Moreover, the clinical significance of CD30 expression for predicting prognosis in ENKTL has been unclear. Here, we measured CD30 expression to evaluate its prognostic value in ENKTL.

## Methods

### Patients

Ninety-six patients were selected with pathologically proven ENKTL diagnosed from August 2000–June 2013 at the Sun Yat-Sen University Cancer Center. Histology, immunophenotype and EBV status were reviewed to confirm the diagnosis based on World Health Organization guidelines [14]. The criteria for case inclusion were as follows: (1) histologically confirmed diagnosis of ENKTL; (2) NK/T-cell type proven using immunohistochemistry (IHC), flow cytometry or EBV *in situ* hybridization; (3) no previous malignancy; (4) no previous treatment for lymphoma; and (5) adequate clinical information and follow-up data. Moreover, patients with aggressive NK cell lymphoma/leukemia, peripheral T-cell lymphoma, blastic NK cell lymphoma/leukemia or negative EBV *in situ* hybridization were excluded from the analysis. The clinical data contained the following information: patient demographics, physical examinations, Eastern Cooperative Oncology Group performance status (ECOG PS), B symptoms, primary site, involved sites, serum β2 microglobulin (β2 M), serum LDH, bone marrow examination, endoscopic examination of the nasal and oral cavity, computed tomography (CT) or magnetic resonance imaging (MRI) of the involved field or whole body positron emission tomography/computed tomography (PET/CT). All patients were staged according to the Ann Arbor staging system, as calculated using the IPI and KPI.

The primary tumor site was classified into two subtypes: upper aerodigestive tract NK/T-cell lymphoma (UNKTL; primary tumors confined to the nasal cavity, nasopharynx, paranasal sinuses, tonsils, hypopharynx and larynx) and extra-UENKTL (EUNKTL; primary tumors at all other sites in the absence of nasal disease) [7, 15]. Primary tumors within the nasal cavity and secondary spread to other organs were regarded as UNKTL. Both the Institutional Review Board and Ethics Committees of Sun Yat-Sen University Cancer Center approved the study. All patients consented to the use of their medical records for research purposes.

### Treatment and response evaluation

Patient treatment strategies were as follows: (1) chemotherapy alone; or (2) chemotherapy followed by involved field radiotherapy (IFRT). The chemotherapy regimens were: (1) EPOCH (etoposide, doxorubicin, vincristine, cyclophosphamide and prednisone); or (2) GELOX (gemcitabine, oxaliplatin and L-asparaginase) or modified GELOX [16]. Patients received at two to six cycles of initial chemotherapy. The IFRT of 36–60 Gy was delivered in daily fractions of 1.8–2.0 Gy (five fractions each week). Treatment response was assessed according to the International Working Group Recommendations for Response Criteria for NHL [17, 18]. Routine follow-up imaging analyses were performed every 3 months for the first 2 years, every 6 months for the next 3 years and yearly thereafter, or whenever clinically indicated.

### IHC for CD30

Representative formalin-fixed, paraffin-embedded tissues obtained from surgical resections or biopsies were submitted for IHC. Four-micrometer-thick sections of paraffin-embedded tissues were cut, placed on slides, deparaffinized in xylene and hydrated in a graded alcohol series. Immunohistochemical staining of CD30 was performed on selected cases using a CD30 antibody (Invitrogen, Carlsbad, CA, USA) incubated at a 1:50 dilution. IHC was performed using a modified avidin-biotin peroxidase complex amplification and detection system. Specimens were analyzed according to the local ethical guidelines and approved study protocols. The percentage of CD30 expression was quantified by determining the amount of positive cells with membrane staining among the total number of tumor cells in the high-power field under high magnification (×400). A semi-quantitative scoring system for CD30 expression was applied using the following categories: (1) “negative”, less than 10% of tumor cells stained; (2) “positive”, 10–50% of tumor cells stained; (3) “strongly positive”, more than 50% of tumor cells with clearly stained cell membranes. Two pathologists (Liu and Zhang) performed all analyses in a single laboratory. The pathologist who performed the cell counts was blinded to the clinical characteristics and survival status.

### Statistical analysis

Overall survival (OS) was determined from the date of diagnosis to the date of death or the last follow-up visit. Progression-free survival (PFS) was measured from the date of diagnosis to the date of disease progression, relapse, death or the date of the last follow-up visit. The relationship of CD30 expression with clinical variables was calculated using the chi-squared test or Fisher’s exact test. The Kaplan-Meier method was used to calculate OS and PFS, and survival curves were compared using the log-rank test. The Cox proportional hazards regression model was used for the multivariate analysis to compare factors proven statistically significant in the univariate analysis. A two-sided p-value of less than 0.05 was considered statistically significant. All analyses were performed using SPSS software (SPSS Standard version 19.0, SPSS, Chicago, IL, USA).

## Results

### Patient characteristics

The main clinical characteristics of the 96 patients are presented in Table 1. The median age was 41 years and ranged from 17 to 89 years. The ratio of males to females was 2:1. Eighty-eight patients (91.7%) had good a ECOG PS of 0–1. The majority of patients initially presented with UNKTL tumors (n = 63, 65.6%) or localized disease (stages I and II; n = 71, 74%). In patients with EUNKTL, primary lesion sites involved the small bowel, colon, lung, skin, testis and soft tissues. Over half of the patients were classified into the low-risk group according to their IPI or KPI score.

**Table 1:** **Clinical characteristics of patients at diagnosis**

Characteristics	Presence	Presence %
Age at diagnosis (years) Median (range)	41 (17–89)	
≤60	83	86.5
>60	13	13.5
Gender		
Male	61	63.5
Female	35	36.5
ECOG PS		
0, 1	88	91.7
≥2	8	8.3
Subtypes		
UNKTL	63	65.6
EUNKTL	33	34.4
B-symptoms	48	50.0
Mass long diameter ≥5 cm	36	37.5
Local tumor invasion	61	63.5
Extranodal sites ≥2	57	59.4
Regional lymphadenopathy	51	53.1
Elevated serum LDH	41	42.7
Elevated serum β_2_ M*	40	67.8
Ann Arbor stage		
I, II	71	74.0
III, IV	25	26.0
IPI score		
0, 1	58	60.4
≥2	38	39.6
KPI score		
0, 1	49	51.0
≥2	47	49.0
CD30 expression		
Positive	30	31.2
Negative	66	68.8
Treatment		
Chemotherapy	35	36.5
Chemotherapy + radiotherapy	61	63.5

Abbreviations: *ECOG PS* Eastern Cooperative Oncology Group performance status, *UNKTL* upper aerodigestive tract NK/T-cell lymphoma, *EUNKTL* extra-upper aerodigestive tract NK/T-cell lymphoma, *LDH* lactate dehydrogenase, *β*_*2*_ *M* Beta-2 microglobulin, *IPI* International Prognostic Index, *KPI* Korean Prognostic Index.

*Serum β_2_ M was detected in 59 cases.

### Correlation between CD30 expression and clinical features

**Figure 1:**
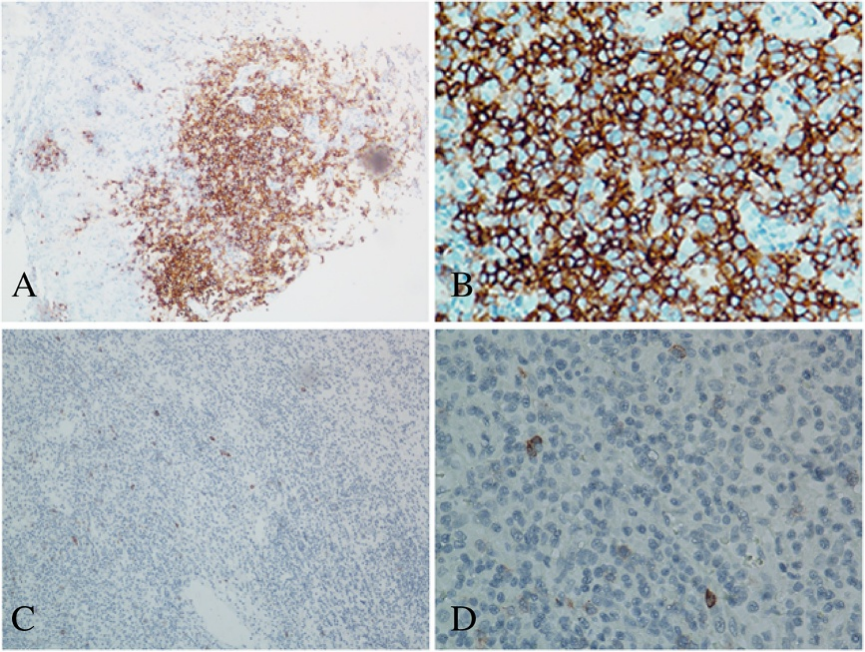
**Immunohistochemical analysis of CD30 expression in extranodal natural killer T-cell lymphomas, nasal type (ENKTL). A** and **B**, representative images of CD30-positive tumor cells showing strong cell membrane staining (brown) (magnification in **A** × 100, **B** × 400); **C** and **D**, representative image of CD30-negative tumor cells showing no membrane staining (magnification in **C** × 100, **D** × 400).

Correlations between CD30 expression and main clinical parameters are summarized in Table 2. CD30 was expressed in 31.2% of ENKTL patients (Figure 1). We observed no association between CD30 expression and other clinical features such as age, gender, local tumor invasion, extranodal sites, ECOG PS, stage, IPI and KPI scores. The CD30-positive group showed statistically significant increases in cases with B symptoms and elevated serum LDH compared with the CD30-negative group (p = 0.028 and p = 0.021, respectively).

**Table 2:** **Clinical characteristics according to CD30-positive versus CD30-negative expression at diagnosis**

Characteristics	CD30-positive	CD30-negative	***P***-value
No. of patients	30	66	
Age at diagnosis (years)			1.000
≤60	26	57	
>60	4	9	
Gender			0.668
Male	20	41	
Female	10	25	
ECOG PS			0.428
0, 1	29	59	
≥2	1	7	
Subtypes			0.434
UNKTL	18	45	
EUNKTL	12	21	
B-symptoms	20	28	0.028
Mass (diameter) ≥5 cm	9	27	0.306
Local tumor invasion	21	40	0.375
Extranodal sites ≥2	20	37	0.327
Regional lymphadenopathy	17	34	0.639
Elevated serum LDH	18	23	0.021
Stage			0.684
I, II	23	48	
III, IV	7	18	
IPI score			0.339
0, 1	16	42	
≥2	14	24	
KPI score			0.057
0, 1	11	38	
≥2	19	28	
Treatment			0.627
Chemotherapy	12	23	
Chemotherapy + radiotherapy	18	43	

Abbreviations: *ECOG PS* Eastern Cooperative Oncology Group performance status, *UNKTL* upper aerodigestive tract NK/T-cell lymphoma, *EUNKTL* extra-upper aerodigestive tract NK/T-cell lymphoma, *LDH* lactate dehydrogenase, *IPI* International Prognostic Index, *KPI* Korean Prognostic Index.

### Treatment response and survival

All 96 patients received chemotherapy; 55 patients received the EPOCH regimen whereas the other 41 patients received the GELOX regimen. Sixty-one patients received chemotherapy followed by IFRT whereas 35 patients received only chemotherapy. No statistical difference was found in the treatment modalities of patients according to CD30-positive vs. CD30-negative expression at diagnosis (p = 0.627).

The treatment response was evaluated in each patient. Results showed that 65 patients (67.7%) and 17 patients (17.7%) achieved complete remission (CR) and partial response (PR), respectively, thus the overall response rate (ORR) was 85.4%. CR rates of patients with CD30-positive and CD30-negative expression were 60.0% (18/30) and 71.2% (47/66), respectively (p = 0.276).

Within a median follow-up time of 31 months (5–152), the 5-year OS and PFS rates were 53.9% (95% CI, 35.0–69.8%) and 42.1% (95% CI, 27.6–56.6%), respectively. Patients who were CD30-positive tended to have shorter OS (5-year OS, 34.1% vs. 64.4%; p = 0.002) and PFS (5-year PFS, 26.0% vs. 66.7%; p < 0.001). In patients who received chemotherapy alone (35 cases, 36.5%), CD30-positivity was associated with shorter OS and PFS (p = 0.037 and p = 0.018, respectively), and in patients who received chemotherapy followed by radiotherapy (61 cases, 63.5%), CD30-positivity at diagnosis was also related to inferior OS and PFS (p = 0.033 and p = 0.005, respectively). For patients in the IPI 0–1 subgroup (58, 60.4%), CD30-positivity was associated with shorter OS and PFS (p = 0.001 and p = 0.002, respectively). Similarly, for patients in the KPI 0–1 subgroup (49, 51.0%), CD30-positivity at diagnosis was also related to inferior OS and PFS (p = 0.018 and p = 0.023, respectively).

### Univariate and multivariate analysis

Univariate analysis showed that B symptoms, two or more extranodal sites, elevated serum LDH, local tumor invasion, advanced stage (III/IV) and CD30-positivity, IPI and KPI could significantly predict shorter OS and PFS. Age and subtype showed statistical significance with OS (p = 0.049 and p = 0.022, respectively), but failed to show prognostic significance for PFS. Clinical factors that were statistically significant predictors of OS and PFS were included in the multivariate analysis. We did not include IPI and KPI values in the multivariate analysis because of their overlap with several other clinical variables. Multivariate analysis revealed that CD30 expression was an independent prognostic factor for OS (response rates (RR) = 3.345; 95% CI, 1.477–7.575; p = 0.004) and PFS (RR = 4.391; 95% CI, 1.940–9.941; p = 0.001). The stage was also an independent prognostic factor for OS (RR = 3.497; 95% CI, 1.314–9.346; p = 0.012) and PFS (RR = 2.841; 95% CI, 1.166–6.945; p = 0.022). However, other factors such as B symptoms, two or more extranodal sites, elevated serum LDH and local tumor invasion failed to be prognostic for OS or PFS (Table 3).

**Table 3:** **Results of univariate and multivariate analyses of prognostic factors for PFS and OS in patients with ENKTL**

Parameter	PFS			OS		
	Univariate analysis	Multivariate analysis		Univariate analysis	Multivariate analysis	
	***P***-value	RR (95% CI)	***P***-value	***P***-value	RR (95% CI)	***P***-value
Age >60 years	0.120			0.049		
Gender, male	0.563			0.733		
ECOG PS ≥2	0.436			0.341		
Subtype, EUNKTL	0.098			0.022		
B symptoms	0.001			0.002		
Mass long diameter >5 cm	0.105			0.174		
Local tumor invasion	0.001			0.003		
Extranodal sites ≥2	0.002			0.007		
Regional lymphadenopathy	0.095			0.301		
Elevated serum LDH	< 0.001			< 0.001		
CD 30 positive	< 0.001	4.391 (1.940–9.941)	0.001	0.002	3.345 (1.477–7.575)	0.004
Stage III, IV	< 0.001	2.841 (1.166–6.945)	0.022	< 0.001	3.497 (1.314–9.346)	0.012
IPI score ≥2	< 0.001			< 0.001		
KPI score ≥2	< 0.001			< 0.001		

Abbreviations: *PFS* progression-free survival, *OS* overall survival, *RR* relative risk, *CI* confidence interval, *ECOG PS* Eastern Cooperative Oncology Group performance status, *EUNKTL* extra-upper aerodigestive tract NK/T-cell lymphoma, *LDH* lactate dehydrogenase, *IPI* International Prognostic Index, *KPI* Korean Prognostic Index.

## Discussion

In this study, the expression for CD30 in ENKTL patients was significantly correlated with B symptoms and elevated serum LDH. Furthermore, although CD30 expression did not appear to affect the response to GELOX or EPOCH chemotherapy, survival analysis indicated that CD30-positive patients had a significantly inferior OS and PFS. According to the Cox regression model that included B symptoms, two or more extranodal sites, elevated serum LDH, local tumor invasion, advanced stage (III/IV) and CD30-positivity, it was concluded that CD30-positivity was an independent prognostic factor for both OS and PFS.

The fact that CD30 was expressed in both tumor cells and certain activated normal lymphoreticular cells implies that it has a general cell-growth or activation role. Hsu *et al.* found a high level of CD30 and CD30L co-expression in H-RS cells, and increased proliferation was noted upon treatment with exogenous CD30L [19]. Primary cutaneous large T-cell lymphomas, which are negative for CD30 originally and develop CD30 secondarily during the course of the disease, present a worse clinical course and have a poor prognosis.

In this present study, we retrospectively analyzed the relationship between CD30 expression and clinicopathological features, and found that CD30-positive expression was more common in patients with B symptoms and elevated LDH levels. As discussed above, binding of CD30 and CD30L can promote proliferation of H-RS cells. This effect may exist in ENKTL, supporting the result that in CD30-positive ENKTL patients, LDH levels, which reflect the speed of tumor cell proliferation, was higher than that in CD30-negative patients. In our study, 31.2% (30/96) of patients showed positive expression of CD30 in ENKTL cells, which corresponded well with the result reported by Junshik *et al.*, in which they found that 36.4% patients (8/22) with ENKTL showed positive expression of CD30 and the prognosis of these patients was inferior to those with negative expression [20].

In the present study, the rates of CR and ORR were not significantly different between the two groups after chemotherapy or radiotherapy, but the survival analysis indicated that the 5-year rates of OS and PFS in the CD30-negative group were both significantly higher than those in the CD30-positive group (64.4% vs. 34.1%, P = 0.002, for 5-year OS; 66.7% vs. 26.0%, P < 0.001, for 5-year PFS). Furthermore, subgroup analysis showed that CD30-negative patients had a better prognosis, regardless of treatment modality (chemotherapy followed by IFRT or chemotherapy alone). Our results were consistent with the study conducted by Junshik *et al.* that showed that patients with CD30 expression had an inferior OS and PFS compared with those without CD30 expression. Nevertheless, CD30-positive patients tended to have a better prognosis in one study (n = 30), while in two other studies performed by Kuo *et al.* (n = 22) [21] and Gaal *et al.* (n = 15) [22], although it appeared that CD30 expression was related to angiodestruction, pleomorphic cell type or thrombus formation, there were no survival differences in terms of CD30 expression. However, in their study, the prognostic significance of CD30 expression was established on the basis of small sample sizes, and one of the studies only referred to NK/T-cell lymphomas presenting on the skin. One other thing to note is the influence of different CD30 cutoff levels on the final result. In our study, CD30 expression was considered positive when more than 10% of tumor cells showed strong membrane staining. Perhaps CD30 cutoff levels, which were different from ours (absolute values not shown in their article), resulted in the contradictory findings mentioned above.

As discussed above, CD30 expression had no effect on the rate of response to treatment, but only affected the long-term survival. The results indicated that CD30 expression in ENKTL cells only promotes cell proliferation without affecting its sensitivity to therapy. Furthermore, the presence of EBV appears to occur more frequently in CD30-positive lymphomas when compared with CD30-negative lymphomas. EBV is known for its ability to upregulate CD30 in EBV-positive lymphoma cell lines. Thus, it is hypothesized that CD30 may be involved in tumor cell growth regulated by EBV in CD30-positive ENKTL and result in a poor prognosis.

**Figure 2:**
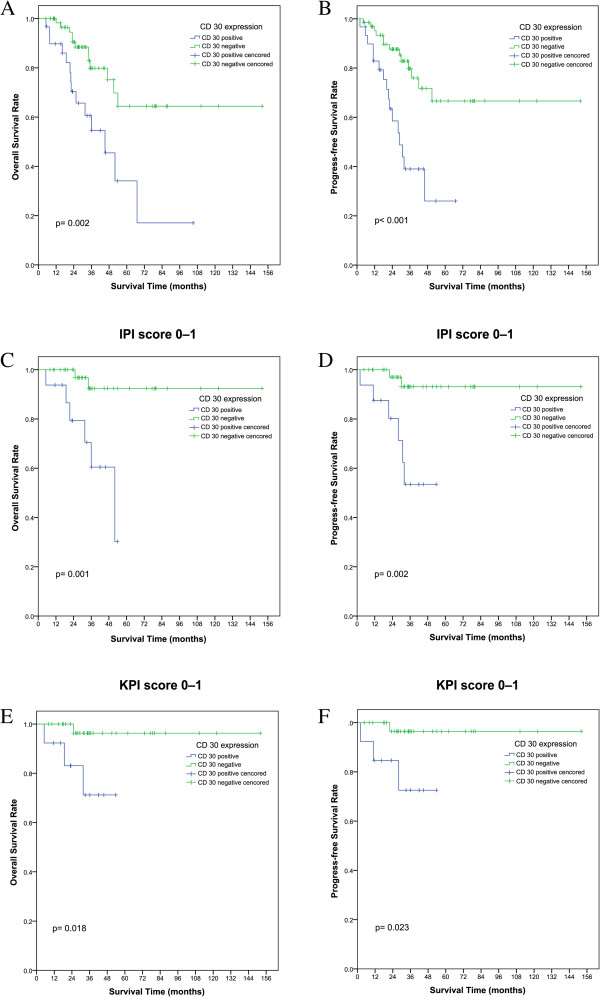
**Overall survival (OS) and progression-free survival (PFS) according to CD30-positive vs. CD30-negative expression at diagnosis in patients with extranodal natural killer T-cell lymphomas, nasal type (ENKTL).** Kaplan-Meier plots of OS **(A)** for all patients and PFS **(B)** for all patients; Kaplan-Meier plots of OS **(C)** and PFS **(D)** for subgroups with low International Prognostic Index (IPI) scores of 0–1; Kaplan–Meier plots of OS **(E)** and PFS **(F)** for subgroups with low Korean Prognostic Index (KPI) scores of 0–1.

Clinically, two major clinical prognostic models are applied in NK/T-cell lymphoma: IPI and KPI. In the present study, univariate analysis showed that the two models were highly prognostic. Distribution of patients within risk groups based on IPI and KPI scores is presented in Table 1. For IPI scores, more than 70% of all cases were in the low-risk category (with no or one adverse factor). The KPI model showed a better balanced distribution of patients into different risk groups than the IPI model. However, these two prognostic models failed to differentiate patients with different outcomes in the low-risk group. As is depicted in Figure 2, CD30 expression can divide patients with IPI or KPI scores of 0–1 into two subgroups with significant differences in OS and PFS (IPI: P = 0.001 and P = 0.002, respectively; KPI: P = 0.018 and P = 0.023, respectively). Thus, CD30 expression can be a good independent prognostic factor for OS and PFS not only in the entire group of ENKTL patients, but also in those with low-risk IPI scores.

Radiation therapy is widely administered to patients with localized nasal disease, and produces a complete response rate of up to 70% [23]. However, local and systemic failures were observed frequently in patients who receive radiation therapy alone [24]. Therefore, chemotherapy is required in combination with radiotherapy to reduce the risk of recurrence. In the present study, some patients developed primary or secondary resistance to chemotherapy. Thus, novel drugs or treatment regimens are urgently needed. SGN-35, a humanized CD30 antibody coupled to monomethyl-auristatin E, exhibited potent and specific cytotoxicity against CD30-positive cells *in vitro* and *in vivo* [25, 26]. SGN-35 (brentuximab vedotin) was approved for treatment in patients with relapsed Hodgkin lymphoma and relapsed systemic anaplastic large-cell lymphoma. As was demonstrated above, one-third of patients with ENKTL highly expressed CD30. Thus, brentuximab vedotin may also have an effect in ENKTL patients, and needs to be tested in preliminary studies and clinical trials.

## Conclusions

In conclusion, it was found that the expression of CD30 was an independent prognostic factor for both OS and PFS in ENKTL, nasal type. Further investigation is required to provide a better understanding of the mechanisms underlying the association between CD30 and clinical outcome. These results need to be validated in prospective trials and may support the incorporation of anti-CD30-targeted therapy into current treatment strategies against ENKTL.

## References

[1] Lim MS, de Leval L, Quintanilla-Martinez L (2009). Commentary on the 2008 WHO classification of mature T- and NK-cell neoplasms. J Hematop.

[2] Vose J, Armitage J, Weisenburger D (2008). International peripheral T-cell and natural killer/T-cell lymphoma study: pathology findings and clinical outcomes. J Clin Oncol.

[3] Au WY, Ma SY, Chim CS, Choy C, Loong F, Lie AK, Lam CC, Leung AY, Tse E, Yau CC, Liang R, Kwong YL (2005). Clinicopathologic features and treatment outcome of mature T-cell and natural killer-cell lymphomas diagnosed according to the world health organization classification scheme: a single center experience of 10 years. Ann Oncol.

[4] Li YX, Liu QF, Fang H, Qi SN, Wang H, Wang WH, Song YW, Lu J, Jin J, Wang SL, Liu YP, Lu N, Liu XF, Yu ZH (2009). Variable clinical presentations of nasal and Waldeyer ring natural killer/T-cell lymphoma. Clin Cancer Res.

[5] Kwong YL, Kim WS, Lim ST, Kim SJ, Tang T, Tse E, Leung AY, Chim CS (2012). SMILE for natural killer/T-cell lymphoma: analysis of safety and efficacy from the Asia lymphoma study group. Blood.

[6] Wang L, Xia ZJ, Huang HQ, Lu Y, Zhang YJ (2012). Cyclophosphamide, doxorubicin, vincristine, and prednisone (CHOP) in the treatment of stage IE/IIE extranodal natural killer/T cell lymphoma, nasal type: 13-year follow-up in 135 patients. Int J Hematol.

[7] Lee J, Suh C, Park YH, Ko YH, Bang SM, Lee JH, Lee DH, Huh J, Oh SY, Kwon HC, Kim HJ, Lee SI, Kim JH, Park J, Oh SJ, Kim K, Jung C, Park K, Kim WS (2006). Extranodal natural killer T-cell lymphoma, nasal-type: a prognostic model from a retrospective multicenter study. J Clin Oncol.

[8] Huang JJ, Jiang WQ, Lin TY, Huang Y, Xu RH, Huang HQ, Li ZM (2011). Absolute lymphocyte count is a novel prognostic indicator in extranodal natural killer/T-cell lymphoma, nasal type. Ann Oncol.

[9] Zhang J, Wang WD, Geng QR, Wang L, Chen XQ, Liu CC, Lv Y (2014). Serum levels of interleukin-9 correlate with negative prognostic factors in extranodal NK/T-cell lymphoma. PLoS One.

[10] Li YJ, Li ZM, Xia Y, Huang JJ, Huang HQ, Xia ZJ, Lin TY, Li S, Cai XY, Wu-Xiao ZJ, Jiang WQ (2013). Serum C-reactive protein (CRP) as a simple and independent prognostic factor in extranodal natural killer/T-cell lymphoma, nasal type. PLoS One.

[11] Durkop H, Latza U, Hummel M, Eitelbach F, Seed B, Stein H (1992). Molecular cloning and expression of a new member of the nerve growth factor receptor family that is characteristic for Hodgkin's disease. Cell.

[12] Farrah T, Smith CA (1992). Emerging cytokine family. Nature.

[13] Gruss HJ, Boiani N, Williams DE, Armitage RJ, Smith CA, Goodwin RG (1994). Pleiotropic effects of the CD30 ligand on CD30-expressing cells and lymphoma cell lines. Blood.

[14] Swerdlow S, Campo E, Harris N, Jaffe E, Pileri S, Stein H, Thiele J, Vardiman J (2008). WHO Classification of Tumours of Haematopoietic and Lymphoid Tissues.

[15] Lee J, Kim WS, Park YH, Park SH, Park KW, Kang JH, Lee SS, Lee SI, Lee SH, Kim K, Jung CW, Ahn YC, Ko YH, Park K (2005). Nasal-type NK/T cell lymphoma: clinical features and treatment outcome. Br J Cancer.

[16] Wang L, Wang Z, Chen XQ, Li YJ, Wang KF, Xia YF, Xia ZJ (2013). First‒line combination of gemcitabine, oxaliplatin, and L‒asparaginase (GELOX) followed by involved‒field radiation therapy for patients with stage IE/IIE extranodal natural killer/T‒cell lymphoma. Cancer.

[17] Cheson BD, Horning SJ, Coiffier B, Shipp MA, Fisher RI, Connors JM, Lister TA, Vose J, Grillo-Lopez A, Hagenbeek A, Cabanillas F, Klippensten D, Hiddemann W, Castellino R, Harris NL, Armitage JO, Carter W, Hoppe R, Canellos GP (1999). Report of an international workshop to standardize response criteria for non-Hodgkin's lymphomas. NCI sponsored international working group. J Clin Oncol.

[18] Grillo-Lopez AJ, Cheson BD, Horning SJ, Peterson BA, Carter WD, Varns CL, Klippenstein DL, Shen CD (2000). Response criteria for NHL: importance of ‘normal’ lymph node size and correlations with response rates. Ann Oncol.

[19] Hsu PL, Hsu SM (2000). Autocrine growth regulation of CD30 ligand in CD30-expressing reed-Sternberg cells: distinction between Hodgkin's disease and anaplastic large cell lymphoma. Lab Invest.

[20] Hong J, Park S, Baek HL, Jung JH, Kang IG, Sym SJ, Park J, Ahn JY, Cho EK, Kim ST, Shin DB, Lee JH (2012). Tumor cell nuclear diameter and CD30 expression as potential prognostic parameter in patients with extranodal NK/T-cell lymphoma, nasal type. Int J Clin Exp Pathol.

[21] Kuo TT, Shih LY, Tsang NM (2004). Nasal NK/T cell lymphoma in Taiwan: a clinicopathologic study of 22 cases, with analysis of histologic subtypes, Epstein-Barr virus LMP-1 gene association, and treatment modalities. Int J Surg Pathol.

[22] Gaal K, Sun NC, Hernandez AM, Arber DA (2000). Sinonasal NK/T-cell lymphomas in the United States. Am J Surg Pathol.

[23] Koom WS, Chung EJ, Yang WI, Shim SJ, Suh CO, Roh JK, Yoon JH, Kim GE (2004). Angiocentric T-cell and NK/T-cell lymphomas: radiotherapeutic viewpoints. Int J Radiat Oncol Biol Phys.

[24] Kim GE, Cho JH, Yang WI, Chung EJ, Suh CO, Park KR, Hong WP, Park IY, Hahn JS, Roh JK, Kim BS (2000). Angiocentric lymphoma of the head and neck: patterns of systemic failure after radiation treatment. J Clin Oncol.

[25] Francisco JA, Cerveny CG, Meyer DL, Mixan BJ, Klussman K, Chace DF, Rejniak SX, Gordon KA, DeBlanc R, Toki BE, Law CL, Doronina SO, Siegall CB, Senter PD, Wahl AF (2003). cAC10-vcMMAE, an anti-CD30-monomethyl auristatin E conjugate with potent and selective antitumor activity. Blood.

[26] Okeley NM, Miyamoto JB, Zhang X, Sanderson RJ, Benjamin DR, Sievers EL, Senter PD, Alley SC (2010). Intracellular activation of SGN-35, a potent anti-CD30 antibody-drug conjugate. Clin Cancer Res.

[CR27] The pre-publication history for this paper can be accessed here:http://www.biomedcentral.com/1471-2407/14/890/prepub

